# 

**Published:** 1790

**Authors:** 


### CATALOGUE OF BOOKS.

1. AN Account* of an Epidemic Fever; with a few Remarks on the Treatment of Fevers in general. By *William May*, M. D. Extra-Licentiate of the College of Phyſicians, London, and Phyſician at Maidſtone. 8vo. Maidſtone, 1790.

2. Reports of the Royal Humane Society; with an Appendix of miſcellaneous Obſerva-

* The Work here announced is a republication of an account of an epidemic fever in Cornwall, inſerted in the tenth Volume of this Journal, with ſome additional obſervations which the later experience of the ingenious author has ſuggeſted.

tions on the Subject of fufpended Animation: for the Years 1787, 1788, and 1789. 8vo. *Cadell*, London, 1790.

3. An Effay on the Prefervation of the Health of Perfons employed in Agriculture, and on the Cure of the Difeafes incident to that Way of Life. By *William Falconer*, M. D. F. R. S. and Phyfician to the Bath Hofpital. 8vo. Bath, 1789.

4. Thoughts upon the Means of preferving the Health of the Poor, by Prevention and Suppreffion of Epidemic Fevers. Addreffed to the Inhabitants of the Town of Manchefter, and of the feveral populous trading Towns furrounding and connected with it. By the Rev. Sir *W. H. Clerke*, Bart. Rector of Bury in the County of Lancafter. 8vo. *Johnfon*, London, 1790.

5. Hiftorical and Biographical Sketches of the Progrefs of Botany in England, from its Origin to the Introduction of the Linnæan Syftem. By *Richard Pulteney*, M. D. F. R. S. 2 Vols. 8vo. *Cadell*, London, 1790.

6. A Treatife on the Typhus Fever; publifhed for the Benefit of eftablifhing a Lying-in Hofpital in Baltimore. By *George Buchanan*, M. D. Prefident of the Royal Phyfical Society of of Edinburgh, and Member of the American Philoſophical Society, &c. 12mo. *Dilly*, London, 1789.

7. The Hiſtory and chemical Analyſis of the Mineral Water lately diſcovered in the City of Glouceſter; the various Diſeaſes to which it is applicable conſidered; and the neceſſary Regulations for drinking it with Succeſs aſcertained. By *John Hemming*, M.D. Phyſician to the Offulſton Diſpenſary, &c. 8vo. *Hookham*, London, 1789.

8. Plan for eſtabliſhing an Inſtitution to cultivate and teach Veterinary Medicine. 8vo. London, 1790.

9. Practical Obſervations upon Thorn Wounds, punctured Tendons, and ligamentary Lameneſs in Horſes; with experimental Inſtructions for their Treatment and Cure. Illuſtrated by a Recital of Caſes, interſperſed with a Variety of uſeful Remarks; to which is added a ſucceſsful Method of treating the Canine Species in that deſtructive Diſeaſe called the Diſtemper: the Whole forming a Supplement to the Gentleman's Stable Directory. By *William Taplin*, Surgeon. 8vo. *Kearſley*, London, 1790.

10. Thoughts and Obſervations on the Nature and Uſe of Dr. James's Powder in the Prevention vention and Cure of Diseases. By a Gentleman of the Faculty. 8vo. *Scatchard*, London, 1790.

11. Dissertatio Medica Inauguralis de Rheumatismo acuto; Auctore *Gulielmo Brooke*, Hiberno. 8vo. Edinburgi, 1789.

12. Dissertatio Medica Inauguralis de Halitu cuticulari; Auctore *Georgio Cayley*, Petropolitano. 8vo. Edin. 1789.

13. Dissertatio Medica Inauguralis de Ictero; Auctore *Joanne Cox*, Anglo. 8vo. Edin. 1789.

14. Dissertatio Medica Inauguralis de Typho; Auctore *Alex. Schaw Field*, Virginiensi. 8vo. Edin. 1789.

15. Dissertatio Medica Inauguralis de Dysenteria; Auctore *Joanne Going*, Hiberno. 8vo. Edin. 1789.

16. Dissertatio Medica Inauguralis quædam de Frigoris generalioribus in corpora Viva effectibus, ejusque usu in Morbis febrilibus fugandis, complectens; Auctore *Jacobo Holman*, Anglo. 8vo. Edin. 1789.

17. Dissertatio Medica Inauguralis de Scrofula; Auctore *Gulielmo Houghton*, Anglo. 8vo. Edin. 1789.

18. Dissertatio Medica Inauguralis de Ictero; Auctore *Georgio Maxwell*, Hiberno. 8vo. Edin. 1789.

19. Dissertatio Medica Inauguralis de Puerperarum Febre; Auctore *Joanne Pentland*, Hiberno. 8vo. Edin. 1789.

20. Dissertatio Medica Inauguralis de Adipe; Auctore *Josepho Redhead*, Antiguensi. 8vo. Edin. 1789.

21. Dissertatio Medica Inauguralis de Febre Puerperarum; Auctore *Georgio Pitt Stevenson*, Marylandico. 8vo. Edin. 1789.

22. Dissertatio Medica Inauguralis de Hypochondriasi; Auctore *Roberto Wightman*, Hiberno. 8vo. Edin. 1789.

23. Dissertatio Medica Inauguralis de Dysenteria; Auctore *Jacobo Box Young*, Georgiensi. 8vo. Edin. 1789.

24. Dissertatio Medica quædam de Hæmorrhagia Uterina gravidis superveniente complectens; Auctore *Joanne Healy*, Hiberno. 8vo. Glasguæ, 1789.

25. *Jo. Frid. Blumenbachii*, Prof. Med. Ordin. M. Britann. R. a Consil. Aul. Societ. R. Scient. Gotting. Aliarumque Membri, Decas Collectionis suæ Craniorum Diversarum Gentium illustrata.

luſtrata. 4to. Gottingæ, 1790. c. Tab. æneis x.

26. *Antonii Jacobi Van Doeveren*, Medicinæ Doctoris, Obſervationes * Pathologico-Anatomicæ. 4to. Lugduni Batavorum, 1789. c. Tab. æn. iii.

27. *Abrahami Van Stipriaan*, Med. Doct. Obſervationes Chemicæ de quibuſdam ſalibus eſſentialibus Vegetabilium. 4to. Lugduni Batavorum, 1788.

28. Diſſertatio Medico-Chemica Inauguralis de Alkali Volatili; Auctore *Thoma Tomſon*, Anglo. 4to. Lugd. Batav. 1788.

29. Diſſertatio Medico-practica Inauguralis de Colica Pictonum; Auctore *Moſes Rodrigues*, Bayona-Gallo. 4to. Lugd. Batav. 1788.

30. Tentamen Medicum Inaugurale de depellendam inſalubritatis calumniam de regione Zelandica; Auctore *Roberto Carolo Ermerins*, Sluſa-Flandro. 4to. Lugd. Bat. 1788.

